# Correction to ‘Starvation differentially affects gene expression, immunity and pathogen susceptibility across symbiotic states in a model cnidarian’ (2024), by Valadez-Ingersoll *et al.*

**DOI:** 10.1098/rspb.2024.1198

**Published:** 2024-06-19

**Authors:** Maria Valadez-Ingersoll, Pablo J. Aguirre Carrión, Caoimhe A. Bodnar, Niharika A. Desai, Thomas D. Gilmore, Sarah W. Davies

*Proc. R. Soc. B* **291**, 20231685 (Published online 28 February 2024). (http://doi.org/10.1098/rspb.2023.1685)

Figure 1D has three incorrect numbers. The number 4858 should be changed to **4860**; the number 1724 should be changed to **1716**. The number 2585 should be changed to **2285**.

Additionally, the number 4858 needs to be changed to **4860** in the Results section a, and twice in the second paragraph of the Discussion. The number 1724 needs to be changed to **1716** in the Results section a, once in the second paragraph of the Discussion, and once in the third paragraph of the Discussion.

